# O Desafio da Ecocardiografia na Avaliação Acurada do Ventrículo Direito e Insuficiência Pulmonar

**DOI:** 10.36660/abc.20210744

**Published:** 2021-10-06

**Authors:** Claudia R. Pinheiro de Castro Grau

**Affiliations:** 1 Instituto do Coração Faculdade de Medicina Universidade de São Paulo São PauloSP Brasil Instituto do Coração da Faculdade de Medicina da Universidade de São Paulo, São Paulo, SP – Brasil; 2 Grupo Fleury São PauloSP Brasil Grupo Fleury, São Paulo, SP – Brasil; 3 Hospital São Luiz Itaim Rede Dor São PauloSP Brasil Hospital São Luiz Itaim, Rede Dor, São Paulo, SP – Brasil

**Keywords:** Cardiopatias Congênitas, Função Ventricular Direita, Insuficiência da Valva Pulmonar, Ressonância Nuclear Magnética, Tetralogia de Fallot/cirurgia

O reconhecimento atual da importância do papel do ventrículo direito (VD) na evolução e prognóstico de várias condições cardiovasculares é indiscutível e a avaliação adequada da sua dimensão e função tem impacto direto no manejo clínico.

A insuficiência pulmonar (IP) é comum após a correção da tetralogia de Fallot (POTF) e após a abordagem de cardiopatias congênitas com obstrução pulmonar, sendo um importante determinante na evolução devido às suas consequências, causando dilatação e disfunção do VD, intolerância ao exercício, maior risco de arritmias e morte súbita.^1^

Os métodos de multimodalidade de imagem são os pilares para a avaliação destes pacientes, sendo incontestável a relevância da ressonância nuclear magnética (RNM) na quantificação da IP e avaliação do VD, enfatizando suas limitações relacionadas à acessibilidade, custo e necessidade de sedação, principalmente nas crianças.^2^ A contribuição da ecocardiografia na prática clínica é inegável, apesar das dificuldades para quantificação da IP e a avaliação do VD, considerados grandes desafios que tem incentivado um número crescente de estudos.^3 , 4^

Estudos para validação da quantificação da IP pelo ecocardiograma (2D) demonstram tendência a superestimar o valor quando comparado à RNM.^5^ A maior eficácia para quantificação pelo 2D é determinada pela análise conjunta de diversos índices estabelecidos na literatura.^6^ Mercer-Rosa et al.,^5^ evidenciaram correlação moderada entre a relação da integral tempo-velocidade do fluxo pulmonar na diástole e sístole e a fração regurgitante pela RNM, estabelecendo um valor de corte para estratificar o grau importante sugerindo a incorporação deste índice aos demais parâmetros para maior eficácia.

Em relação à avaliação da dimensão do VD, a literatura mostra fraca concordância entre as medidas lineares avaliadas pelo 2D e a RNM, com tendência do 2D a subestimar.^7^ Entretanto, dada a geometria complexa do VD que impede a visibilização em um único plano, é notório que a medida linear isolada compromete a avaliação. A inclusão da planimetria da área diastólica final, associada as medidas bidimensionais nos planos apical de quatro câmaras e paraesternal eixo curto para avaliação da via de saída contribuem com a precisão. Nos estudos realizados por ambos os autores, Shiran et al.,^8^ e Alghamdi et al.,^3^ em adultos com hipertensão pulmonar e crianças portadoras de cardiopatia congênita foi possível estimar os volumes por meio da medida da área diastólica indexada no 2D com boa correlação.

A ecocardiografia tridimensional (3D) tem contribuído para avaliação volumétrica e funcional, possibilita a análise de todos os segmentos com ausência de contraindicação, mas requer equipamento específico não disponível em todos os serviços. Há significativa correlação entre o 3D e a RNM e certa tendência do 3D a subestimar os volumes quando há dilatação acentuada.^9^ Os valores de referência em crianças e adultos estão disponíveis na literatura.^10 , 11^

A avaliação da função sistólica do VD é baseada sempre na abordagem integrada da análise qualitativa com parâmetros quantitativos que refletem a função global, variação fracional da área (FAC) e longitudinal, medida da excursão sistólica do anel tricúspide (TAPSE ) e velocidade sistólica pelo Doppler tecidual.^11^ A interpretação do valor do TAPSE como medida isolada nas cardiopatias congênitas deve ser cautelosa, pois reflete apenas o encurtamento longitudinal e sofre a influência da pericardiotomia,^12^ enquanto o valor do FAC, índice de avaliação global apresenta boa correlação com a fração de ejeção, apesar das dificuldades inerentes ao delineamento da cavidade ventricular.^8^

A avaliação do *strain* longitudinal de pico sistólico tem possibilitado maior acurácia na análise funcional do VD, com a peculiaridade de possibilitar a detecção precoce da disfunção e preceder a redução da fração de ejeção.^13^ No POTF a incorporação do *strain* aos demais parâmetros convencionais de avaliação funcional tem contribuído com a acurácia, sendo um avanço no desafio da ecocardiografia para avaliação do VD.^1 - 14^

No artigo^15^ os autores evidenciaram baixa concordância entre o 2D e a RNM na avaliação da dimensão e função do VD, além do grau de IP e notaram que o 2D subestimou a dimensão e superestimou a função e grau de IP. Importante salientar que no estudo a medida da área e da via de saída no plano eixo curto não foram abordadas, o que pode ter colaborado para o valor subestimado pelo 2D. Entretanto, um achado importante foi a triagem da maioria dos casos com dilatação moderada a importante, apontando a relevância na prática clínica do 2D no seguimento e na indicação precisa da RNM. No que diz respeito ao valor superestimado da função pelo 2D, poderíamos especular que a não inclusão do *strain* bidimensional que possibilita a detecção precoce da disfunção, previamente a redução da fração de ejeção possa ter corroborado o resultado.

Neste contexto vale ressaltar que a atitude clínica para a tomada de decisão depende da abordagem integrada entre os dados clínicos e os diferentes métodos de imagem, que são complementares, buscando desse modo a melhor perspectiva para o paciente.
