# Supplementary material for: O Desafio da Ecocardiografia na Avaliação Acurada do Ventrículo Direito e Insuficiência Pulmonar
Source: Arq Bras Cardiol. 2021 Oct 6;117(4):699–700. [Article in Portuguese] doi: 10.36660/abc.20210744 (PMC8528367; doi:10.36660/abc.20210744)
Supplement: Translation

# Translation: The Challenge of Echocardiography in the Accurate Assessment of the Right Ventricle and Pulmonary Insufficiency

**Authors:** Claudia R. Pinheiro de Castro Grau

**Affiliations:** 1 Brazil Instituto do Coração da Faculdade de Medicina da Universidade de São Paulo, São Paulo, SP – Brazil; 2 Brazil Grupo Fleury, São Paulo, SP – Brazil; 3 Brazil Hospital São Luiz Itaim, Rede Dor, São Paulo, SP – Brazil

**Keywords:** Heart Defects,Congenital, Ventricular Function, Right, Pulmonary Valve Insufficiency, Magnetic Resonance Spectroscopy/methods, Tetralogy of Fallot/surgery

The current recognition of the importance of the right ventricle (RV) role in the evolution and prognosis of various cardiovascular conditions is indisputable and the proper assessment of its dimension and function has a direct impact on clinical management.

Pulmonary insufficiency (PI) is common after tetralogy of Fallot repair (POTF) and approach of congenital heart disease with pulmonary obstruction, being is an important determinant of outcome due to its consequences, causing RV dilation and dysfunction, exercise intolerance, increased risk of arrhythmias and sudden death.^1^

Multimodality imaging methods are the pillars for assessing these patients. The relevance of nuclear magnetic resonance (NMR) in the quantification of PI and evaluation of the RV is unquestionable, emphasizing its limitations related to accessibility, cost and need for sedation, especially in children.^2^ It is undeniable the contribution of echocardiography in clinical practice, despite the difficulties in quantifying PI and evaluating the RV, considered significant challenges that have encouraged a growing number of studies.^3 , 4^

Studies to validate the quantification of IP by echocardiography (2D) tend to overestimate the value compared to MRI.^5^ The greater efficacy for quantification by 2D is determined by the joint analysis of several indices established in the literature.^6^ Mercer-Rosa et al.,^5^ showed a moderate correlation between the the ratio of diastolic and systolic time-velocity integrals and the regurgitant fraction by MRI, establishing a cutoff value to stratify the important degree and suggesting the incorporation of this index to the other parameters for greater efficacy.

Regarding the RV dimension assessment, the literature shows weak agreement between the linear measurements assessed by the 2D and the MRI, with the 2D tending to underestimate.^7^ However, given the complex geometry of the RV that prevents visualization in a single plane, it is clear that the isolated linear measure impair the evaluation. The inclusion of planimetry at the end-diastolic area, associated with two-dimensional measurements in the apical four-chamber and parasternal short axis planes for evaluating the outflow tract, contributes to the accuracy. In the studies carried out by both authors, Shiran et al.^8^ and Alghamdi et al.^3^ in adults with pulmonary hypertension and children with congenital heart disease, it was possible to estimate the volumes by measuring the diastolic area indexed in the 2D with good correlation.

Three-dimensional (3D) echocardiography has contributed to volumetric and functional assessment, enabling the analysis of all segments with no contraindication, but it requires specific equipment not available in all services. There is a significant correlation between 3D and MRI and a certain tendency for 3D to underestimate volumes when there is pronounced dilatation.^9^ Reference values for children and adults are available in the literature.^10 , 11^

The assessment of RV systolic function is always based on the integrated approach of qualitative analysis with quantitative parameters that reflect global function, fractional area change (FAC) and and also longitudinal function, tricuspid annular plane systolic excursion (TAPSE) measurement and systolic velocity by Tissue Doppler.^11^ The interpretation of the TAPSE value as an isolated measure in congenital heart diseases should be cautious, as it only reflects the longitudinal shortening and is influenced by pericardiotomy.^12^ In contrast, the FAC value, global assessment index, has a good correlation with the ejection fraction, despite the difficulties inherent in the delineation of the ventricular cavity.^8^

The evaluation of peak systolic longitudinal strain has enabled greater accuracy in the functional analysis of the RV, with the peculiarity of enabling the early detection of the dysfunction and preceding the reduction of the ejection fraction.^13^ In POTF, the incorporation of strain to other conventional evaluation parameters has contributed to the accuracy, advancing on the echocardiography challenge for RV assessment.^1 - 14^

In the article,^15^ the authors evidenced weak agreement between the 2D and the MRI in evaluating the RV dimension and function, in addition to the degree of PI, and noted that the 2D underestimated the dimension and overestimated the PI function and degree. It is important to note that in the study the measurement of the end-diastolic area and right ventricular outflow tract in paraesternal short-axis were not included, which may have contributed to the underestimated value by 2D. However, an important finding was the screening of most cases with moderate to severe dilation, pointing out the relevance in clinical practice of 2D in the follow-up and the precise indication of MRI. Regarding the overestimated value of the function by 2D, we could speculate that the non-inclusion of the two-dimensional strain that enables the early detection of the dysfunction before the reduction of the ejection fraction may have corroborated the result.

In this context, it is noteworthy that the clinical attitude towards decision-making depends on the integrated approach between clinical data and different imaging methods, which are complementary, thus seeking the best perspective for the patient.
